# One Incremental Stride for Doxycycline, One Substantial Advancement for Thyroid Eye Disease

**DOI:** 10.3390/diagnostics14080791

**Published:** 2024-04-10

**Authors:** Ines Matoc, Kim Kasa, Armin Kasumović, Ante Prpić, Ante Vukojević, Ognjen Zrinšćak, Jelena Škunca Herman, Blanka Doko Mandić, Ivan Sabol, Renata Iveković, Zoran Vatavuk

**Affiliations:** 1 Ophthalmology Department, Sestre milosrdnice Zagreb UHC, 10000 Zagreb, Croatia; inesmatoc@gmail.com (I.M.); kimkasa@yahoo.com (K.K.); kasumovic.armin@gmail.com (A.K.); ap.prpic@gmail.com (A.P.); ante.vukojevic1@gmail.com (A.V.); ozrinscak@yahoo.co.uk (O.Z.); blanka.doko.mandic@gmail.com (B.D.M.); renata.ivekovic@kbcsm.hr (R.I.); zo.vatavuk@gmail.com (Z.V.); 2 Division of Molecular Medicine, Ruđer Bošković Institute (RBI), 10000 Zagreb, Croatia; ivan.sabol@irb.hr

**Keywords:** doxycycline, thyroid eye disease, autoimmune thyroid dysfunction

## Abstract

The aim of this study is to assess the effectiveness of a 12-week doxycycline treatment for thyroid eye disease (TED), an autoimmune condition associated with thyroid dysfunction. In this randomized controlled clinical trial, 82 patients were randomly assigned at a 1:1 ratio to receive doxycycline (50 mg) or to undergo no treatment. Various metrics, including margin reflex distance (MRD1 and MRD2), eyelid aperture, levator muscle function, lagophthalmos, proptosis, ocular motility, diplopia, and Graves’ ophthalmopathy-specific quality-of-life (GO-QOL) scale scoring were assessed. Exclusion criteria were uncontrolled systemic diseases, tetracycline allergies, pregnancy, lactation, or age below 18. The mean age was 51.6 years (SD), 87.8% of participants were female, and all were Caucasians. By week 12, the doxycycline group exhibited a significant improvement rate based on MRD2 (from 4 to 15 participants with physiological findings), clinical activity score (from 7 to 35 participants with non-active disease), and GO-QOL (from 51.22% to 70.73% of participants with a good life quality). Doxycycline showcased anti-inflammatory and immunomodulatory effects in treating TED, suggesting its potential efficacy for TED and other orbit inflammatory conditions. However, these results warrant further validation through future research involving extended follow-up periods and larger cohorts.

## 1. Introduction

Thyroid eye disease (TED) is a complex autoimmune disease associated with thyroid dysfunction. Patients mostly have Graves’ hyperthyroidism, but the disease may as well be seen in euthyroid patients or in those with Hashimoto’s hypothyroidism [1,2,3,4]. It is the most common inflammatory orbital disorder, which causes facial disfigurement and severely impact patients’ quality of life and is the most common cause of unilateral and bilateral proptosis in adults [5]. Proptosis is often cited as <3 mm above the upper limit of normal for race [6]. Other clinical manifestations can vary and may include eyelid swelling and retraction, conjunctival chemosis and injection, strabismus, lagophthalmos with exposure keratopathy, and optic neuropathy [7]. Patients usually complain of eye pain, excessive lacrimation, diplopia, photophobia, and blurry vision [8]. 

The most feared complications of TED are exposure keratopathy with ulceration and dysthyroid optic neuropathy with decreased color vision, visual field defects, and with delayed treatment, permanent optic nerve atrophy and irreversible vision loss [3,9]. The onset of TED occurs typically between 30 and 50 years of age [10], with an incidence of 20–50 per 100,000 people per year [11]. The main risk factors include smoking, female gender, and stress [12,13]. Treatment options vary from only supportive management such as ocular lubricants, sunglasses, prisms, and selenium to oral or intravenous glucocorticoids, orbital irradiation, and biological therapy, depending on disease activity and severity [14,15,16]. A surgical approach to TED is considered if there is proptosis, restrictive strabismus, eyelid retraction, or cosmetic complaints such as periocular fat bags. Based on this, the option of orbital decompression, extraocular muscle surgery, eyelid repositioning, and cosmetic soft tissue remodeling can be considered, but it should be noted that all surgical options have to be provided in the inactive phase of TED [17,18]. 

In the pathogenesis of TED, orbital fibroblasts are considered the key target which may modulate immune responses through the production of cytokines and hyaluronan in response to the activation of shared autoantigens including thyrotropin receptor (TSHR) and insulin-like growth factor-1 receptor (IGF-1R) [19]. Not only autoantigens and TSHR antibodies (TSHRAb) but also T cells play a role in the development of TED [20,21]. 

Doxycycline, a synthetic tetracycline, has demonstrated anti-inflammatory and immunomodulatory effects at doses ranging from 40 mg to 200 mg daily. It has non-antibiotic activities through the suppression of proinflammatory cytokines and chemokines, the downregulation of the protein kinase C pathway, and the inhibition of T cells [19] and phospholipase A2 [22]. Doxycycline combats inflammation by blocking calcium-dependent microtubular accumulation and proliferation of lymphocytes, thus hindering leukocyte movement. It also inhibits nitric oxide synthase (NOS), reducing the production of nitric oxide (NO), a crucial inflammatory signaling molecule [6] and matrix metalloproteinase (MMP) in vitro [23]. 

Regardless of the antibiotic properties achieved at a dose of 200 mg/d, to achieve the anti-inflammatory and immunomodulatory effects of doxycycline, a sub-antimicrobial dose of doxycycline of 40 or 50 mg/d is used [24,25].

In this study, we aimed to investigate the efficacy of 12 weeks of treatment of thyroid eye disease with 50 mg of peroral doxycycline. 

## 2. Materials and Methods

We conducted a randomized clinical trial which included eighty-two sets of eyes of eighty-two age-matched patients with thyroid eye disease. The patients were recruited at a tertiary eye-care center. After detailed information was provided, informed consent was obtained from all subjects involved in the study. This study followed the tenets of the Declaration of Helsinki and all experimental protocols were approved by the Ethics Committee of the University Hospital Centre Sestre milosrdnice (EP-5992/17-1, 13 April 2017).

Participants with uncontrolled systemic diseases or allergy to tetracyclines, and those who were pregnant, lactating, or younger than 18 were excluded.

Participants were eligible for enrollment if they were older than 18 years and were diagnosed as having TED for less than 18 months, defined as meeting at least 2 of the following criteria: (1) concurrent or recently treated immune-related thyroid dysfunction; (2) typical ocular signs; (3) radiographic evidence of TED. 

(1) Immune-related thyroid dysfunction included Graves’ hyperthyroidism, Hashimoto’s thyroiditis, or the presence of circulating thyroid antibodies. (2) Typical ocular signs included one or more of the following: eyelid retraction with temporal flare, proptosis, restrictive strabismus, compressive optic neuropathy, fluctuating eyelid edema and/or erythema, conjunctival edema (chemosis), and/or caruncular edema. (3) Radiographic evidence, via magnetic resonance imaging (MRI) (Siemens, Erlangen, Germany, version VB19A), of TED included enlargement of the inferior, medial, lateral, or superior rectus muscle, with or without enlargement of the levator muscle complex. 

All participants underwent a complete ophthalmic examination that included best-corrected distance visual acuity (BCDVA), a slit-lamp examination, and an intraocular pressure (IOP) measurement (standard Goldmann applanation tonometry), followed by measurements of the margin reflex distance to the upper and lower eyelid (MRD 1 and MRD 2, respectively) (millimeters), eyelid aperture (millimeters), levator palpebrae superioris muscle function (millimeters), lagophthalmos (millimeters), clinical activity score (CAS), proptosis (millimeters, measured with a Hertel exophthalmometer (Oculus, Wetzlar, Germany)), and ocular motility (in four ductions, including upgaze, downgaze, adduction, and abduction), along with diplopia evaluation and modified Graves’ ophthalmopathy-specific quality-of-life (GO-QOL) scale scoring (Appendix A). 

The CAS system was used to assess the activity of TED and was made up of seven components: two symptoms—(1) pain or pressure in a periorbital or retroorbital distribution and (2) pain with upward, downward, or lateral eye movement; and five signs—(1) swelling of the eyelids, (2) redness of the eyelids, (3) conjunctival injection, (4) chemosis, and (5) inflammation of the caruncle or plica. Each component is evaluated as 0 (absent) or 1 (present) where the sum score of 3 or more indicates active disease.

Ocular motility was defined and scored as 1 (performs completely) and 0 (does not perform completely or does not perform at all).

A modified Graves’ ophthalmopathy-specific quality-of-life (GO-QOL) questionnaire was used to evaluate the impact of TED on life quality. The GO-QOL questionnaire is made up of 16 questions about visual function in everyday activities and the psycho-social function of one’s appearance. It was taken as noted below and later modified so that each question could be ranked as 0 (absent) or 1 (present) and validated. The sum of the scores was then calculated and transformed to a scale from 0 (full limitation) to 16 (absent limitation), where the sum of 14 or more was considered a good life quality. 

All examinations were performed by the same observers who were not aware of the treatment status, using the same instruments at each site. 

For each criterion (eyelid aperture, levator palpebrae superioris muscle function, CAS, proptosis, and ocular motility), both eyes were compared and the more severely affected eye was given one point. Based on these measurements, the more severely affected eye was selected as the study eye. In cases of the same summary score, the right eye was chosen. 

Participants were randomly assigned at a ratio of 1:1 to receive doxycycline (50 mg) or to be without treatment for 12 weeks as a control group. A total of 120 tablets of doxycycline were prescribed to participants at baseline and medication compliance was checked during following visits by interviews and counting excess tablets. 

All measurements were taken at baseline, week 4, and week 12. Smoking status was assessed at baseline. Thyroid function assessment was performed at baseline and week 12 and included thyroid peroxidase antibodies (anti TPO), antithyroglobulin antibodies (anti Tg), thyrotropin receptor antibodies (TRAb), thyroid stimulating hormone (TSH), free triiodothyronine (FT3), and free thyroxine (FT4).

Adverse events were recorded in terms of those related to tetracyclines (gastrointestinal discomfort, hepatotoxicity, skin photosensitivity, and pigment accumulation in nails, skin, sclerae, and teeth). Liver function was monitored via liver function tests during follow-up. Participants were advised to avoid direct ultraviolet light.

Patient data and measurements were collected in a Microsoft Excel database. The statistical assessment was performed in Medcalc v22.004 (MedCalc Software Ltd., Ostend, Belgium). The normality of the numerical variables was assessed using the Kolmogorov–Smirnov test. The data were summarized with the mean and standard deviation (SD), median and interquartile range (IQR), or percentages, as appropriate. Differences in numerical variables between treated and untreated patients were assessed with a *t*-test or Mann–Whitney test depending on the normality. Categorical data were assessed with the Chi square test. Patients were also grouped by CAS (≤2 was considered normal and ≥3 was considered active disease), GO-QOL (≥14 was considered good and <14 was considered impaired), and MRD (4.5–5.5 was considered normal and >5.5 was considered abnormal).

To assess time-dependent data across the three weeks of measurements (baseline—0 weeks, 4 weeks, and 12 weeks), the Friedman test was used for treated and untreated patients separately. The paired samples Wilcoxon test was used when there were only 2 timepoints present. Time-dependent paired categorical variables were assessed with the McNemar test for 2 time points and Cochran’s Q test when 3 timepoints were available. *p* values ≤ 0.05 were considered statistically significant.

## 3. Results

During a period of 6 months, patients with TED were enrolled in the study, with a total of 43 eyes assigned to doxycycline and 41 with no therapy. A total of 82 participants completed the 12-week trial. Two participants in the doxycycline group discontinued treatment due to mild adverse effects and were excluded. One case exhibited gastrointestinal discomfort and the other suffered mild skin photosensitivity. 

Table 1. Patients were 51.6 ± 12.7 years in age. A total of 87.8% were female eyes and 100% were Caucasian. There was no significant difference in the demographic information between the treated and untreated patients (Table 1).

Impairment of ocular parameters as well as clinical measurements at baseline and the treatment changes over time are shown in Table 2. After 12 weeks of treatment, a noticeable and statistically significant MRD2, CAS, and GO-QOL improvement was observed in the doxycycline-treated group.

**Figure 1:**
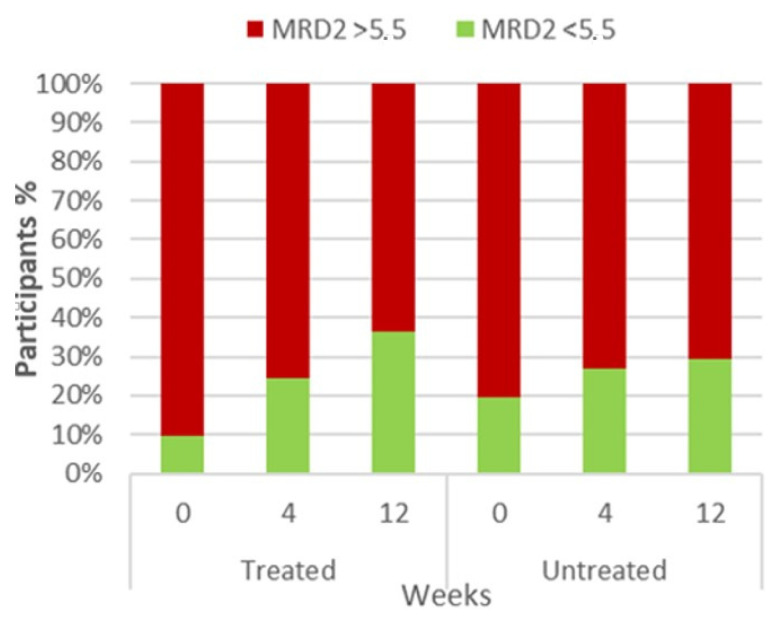
Lower eyelid margin reflex distance (MRD2) in treated vs. untreated patients based on physiological range (**left**) and measurement (**right**).

During the 12-week treatment, there was a significant reduction in the MRD2 measurement in the treated group from an initial median of 7 mm (IQR 6.8–8) down to 6 mm (5–7) at the end of the study, with the change being significant (Friedman test, *p* < 0.0001; Table 2, Figure 1). In the untreated group there was no perceptible change, with the median measurement starting at 7 and remaining at 7 mm (Friedman test, *p* = 0.1939; Table 2, Figure 1). 

More importantly, the MRD2 measurements decreased to the referent range in more treated patients than in untreated patients. In the population treated with doxycycline, at the baseline visit, only 4 participants (9.8%) had an MRD2 score of less than 5.5 mm, which is considered a normal finding, while at the 12-week final visit, 15 of 41 participants (36.6%) had an MRD2 value in the physiological range. On the other hand, 8 untreated participants (19.5%) were within normal limits at baseline and 12 untreated participants (29.3%) were within normal limits at week 12, suggesting less prominent correction of MRD2 (Figure 1). Unlike MRD2, MRD1 did not change meaningfully in either treated or untreated groups.

**Figure 2:**
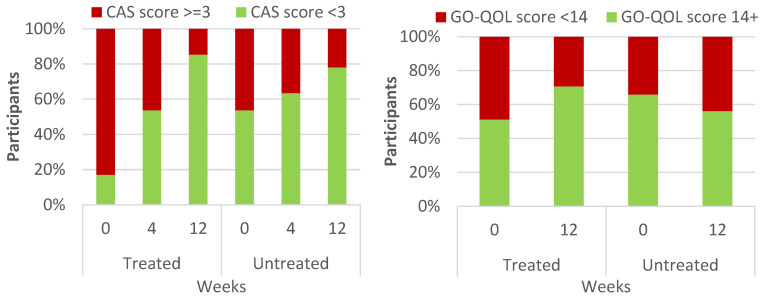
Clinical activity score (**left**) and life quality according to modified Graves’ ophthalmopathy-specific quality-of-life (GO-QOL) questionnaire (**right**) in treated vs. untreated patients at different timepoints.

At the baseline visit, in the treated group, 34 of 41 participants (82.9%) had active disease (CAS ≥ 3) (Table 2, Figure 2). After 12 weeks of doxycycline treatment, only 6 (14.6%) still had active disease. This reduction (28/41 = 68.3% patients with improvement) was highly significant (Cochran’s Q test, *p* < 0.001). In the untreated population, 19 patients started with active disease (46.3%), and 9 of them still had active disease at the end of the study. Most patients did not have a change in status (70.7%), with 1 patient showing a worsening of the CAS score and only 11 patients actually showing improvement (11/41 = 26.8%). The difference in the number of patients exhibiting improvements between the treated and untreated populations was significant (Chi square test, *p* = 0.0007).

Similarly, 20 out of 41 treated patients had some quality of life impairment at baseline (48.8% had a GO-QOL score < 14), which decreased to 12 patients at the end of the study (29.3%) (Table 2, Figure 2). The reduction was significant (McNemar test, *p* = 0.0078). In stark contrast, within the untreated group, there were 14 patients with a QOL impairment (34.1%) at baseline, which increased to 18 patients (43.9%) at the end of the study. Thus, an improvement in the subjective QOL score was seen in 8/41 treated patients (19.5%) but a deterioration was seen in 4/41 (9.8) untreated patients. The differences were statistically significant (Chi square test, *p* = 0.0022).

The number of patients suffering from diplopia or ocular motility impairments also decreased during the study (Table 2). In the case of diplopia, there were 20/41 (48.8) patients showing an improvement in the treated group and only 8/41 (19.5%) in the untreated group, with both groups containing 1 patient each with a deterioration of diplopia. Statistically, the treated group had more cases of improvement (Chi square, *p* = 0.0191). The changes in ocular motility and proptosis measurements were similar in both the treated and untreated groups. The number of patients with abnormal lagophthalmos measurements were too low for a meaningful analysis, with only three cases of 1 mm deviation in the treated group and one case of 1 mm deviation at baseline, which fell to two cases in the treated group and no cases in the untreated group.

## 4. Discussion

In this randomized clinical trial, treatment with doxycycline was shown to be successful in the treatment of TED and is a promising therapy to reduce both symptoms and signs of the disease. 

Current therapies for mild disease include observation and local treatment, which often do not alleviate the disfigurement and negative impact of the disease on the quality of life of these patients. Since there are no clinical guidelines for the treatment of TED, it is necessary to find effective and safe treatment which is also relatively affordable. 

In our study, CAS, as an objective indicator of active disease, showed significant improvement in the group of patients treated with doxycycline compared to the control group; modified GO-QOL results also improved in the treated group.

Adverse events were reported from two participants in relation to doxycycline and they included one case of gastrointestinal discomfort and another of mild skin photosensitivity. The low rate of side effects could be related to the low doses and short duration of doxycycline administration in addition to the well-conducted patient warnings before starting therapy. These results were consistent with previous studies [26,27] using doxycycline at doses up to 200 mg for up to 12 months. However, it should be emphasized again that a large trial sample size with a longer follow-up period is needed to draw a conclusion about the safety of this therapy [28].

The exact mechanism by which doxycycline treats TED is not completely understood, but it is believed to involve anti-inflammatory effects. Doxycycline has been shown to inhibit matrix metalloproteinases (MMPs), enzymes that play a role in tissue remodeling and inflammation. By reducing MMP activity, doxycycline may help control the inflammatory process associated with TED, ultimately alleviating symptoms and preventing further damage. It seems that a study by Kapelko-Slowik et al. [29] suggests that elevated serum concentrations of MMP-2 and MMP-9 are associated with Graves’ disease (GD), Graves’ ophthalmopathy (GO), and hyperthyroidism. Additionally, in patients with TED, there is a notable positive correlation between MMP-9 and serum TSHRAb concentrations. The conclusion drawn is that MMP-9 could serve as an indicator for differentiating and assessing disease severity in GD patients with or without TED. 

The next pertinent mechanism of action for doxycycline involves its interaction with nitric oxide (NO), a signaling molecule participating in a diverse array of physiological activities within the human body influencing inflammation, immunity, and hormone secretion. It is synthesized by enzyme nitric oxide synthase (NOS), from the amino acid L-arginine. The connection between NO and the thyroid is significant, with implications for conditions like hypothyroidism and hyperthyroidism. In hypothyroidism, NO is observed to play a crucial role in tissue damage. NO may also affect the response to hyperthyroidism, potentially causing cardiovascular dysfunction, with gender possibly playing a role. These studies underscore NO’s regulatory impact on various physiological processes in the thyroid [30,31,32]. In addition, Kayser et al. [33] found that NOS expression was much higher in thyroid tumor tissues.

Nitric oxide synthase plays a role in TED by contributing to the regulation of vascular function and inflammation. In TED, there is an increase in NOS activity, leading to elevated nitric oxide production. NO, in turn, affects blood vessel dilation and immune responses, potentially contributing to the eye symptoms associated with TED, such as proptosis and inflammation. The exact mechanisms are complex and involve interactions with the immune system, but NOS and NO are considered factors in the pathophysiology of thyroid eye disease [34,35]. 

Research by Amin AR. et al. [36] reveals a mechanism of action of tetracyclines, demonstrating effects on nitric oxide synthases beyond their antimicrobial action. They inhibit NOS expression and NO production, suggesting potential therapeutic applications [37]. This inhibition of NOS activity by tetracyclines may have additional protective effects by modulating NO production, which is known to have effects on many pathological conditions and manifestations [38]. Tetracyclines inhibit NOS activity by downregulating NOS mRNA expression, leading to decreased NOS protein expression and activity [36]. This attribute of tetracyclines positions them as viable candidates for modulating NO levels in various pathological conditions.

Another potential mechanism by which doxycycline may affect TED involves calcium channels and lymphocyte proliferation. In the normal thyroid, calcium signaling assumes a crucial role, and accumulating evidence suggests its involvement in the progression of thyroid cancer [39]. Calcium stimulates nitric oxide (NO) synthase and increases NO production [40]. Increased calcium levels can trigger microtubular assembly, affecting various cellular processes, including inflammation and immune response. In TED, calcium-dependent signaling pathways may contribute to the abnormal immune response, leading to lymphocytic infiltration and proliferation in the orbital tissues. This immune response may be a key aspect of the inflammatory process observed in thyroid eye disease, contributing to symptoms such as proptosis and orbital tissue expansion [39]. 

In 2015, Lin M. et al. [28] reported their study involving 13 participants with TED who received identical doses of doxycycline for the same period as in our study. Treatment response was followed for 24 weeks and they found overall improvement in 61.5% (8 out of 13 patients), mostly with improvements in CAS, eyelid aperture, and ocular motility. When we analyze our results, there is overall improvement mostly in MRD2 (from 4 to 15 participants), CAS (from 7 to 35 participants), and modified GO-QOL score (from 51.22% to 70.73% of participants). Also, they observed treatment response after 24 weeks and included only patients with Graves’ orbitopathy, while we ended our observation after 12 weeks and included patients with Hashimoto’s thyroiditis and the presence of circulating thyroid antibodies as well. In any case, the conclusion is similar in that a sub-antimicrobial dose of doxycycline appears to be effective and safe in the treatment of active thyroid orbitopathy.

Considering other therapeutic options for TED, in 2020, a group of Italian authors reviewed the literature on the possible beneficial effects and clinical use of selenium in the management of patients with TED. Studies that were carried out until then regarding the use of antioxidant agents, especially selenium, provided conflicting results on the possibility of a new therapeutic approach [41,42]. 

For example, in 2011, the European Group on Graves’ Orbitopathy (EUGOGO) performed a randomized, placebo-controlled, multicenter, clinical trial in European countries in which TED improved in 61% of patients treated with selenium and in 36% of patients receiving placebo, whereas the eye disease worsened in 7 and 26% of patients in the selenium and placebo group. In our study, CAS spontaneously decreased in 11 of 41 participants, and MRD2 recovered in 5 of 41, while subjective quality of life according to GO-QOL remained the same or even worsened in all subjects. 

In 2022, Romanian authors presented a rare case of a middle-aged male patient diagnosed with Graves’ orbitopathy who had an atypical rapid unilateral onset, with the same symptoms developing in the right eye within a short period of time [43]. They treated him with doxycycline, as they reported, due to its efficiency in the treatment of inflammatory ocular conditions by inhibiting the synthesis and the release of corneal MMPs but also included initial intravenous methylprednisolone at 500 mg weekly for 6 weeks, and then reduced to 250 mg weekly for 6 weeks with topical broad-spectrum antibiotics and preservative-free lubricants. At the sixth month follow-up, the ophthalmologic examination revealed an improvement in exophthalmos in both eyes, but since the patient received different types of therapy, the improvement cannot be linked specifically to doxycycline.

Pan Y. et al. [44] conducted a placebo-controlled, multicenter, randomized, double-masked trial and reported by the end of 2022 that at week 12, the primary outcome (assessed by a composite indicator: eyelid aperture, proptosis, ocular motility, and GO-QOL) showed improvement rates in symptoms associated with mild TAO of 38.0% in the doxycycline group and 16.0% in the placebo group. Secondary outcomes, including the analysis of individual clinical symptoms (eyelid aperture, proptosis, eye muscle motility, upper and lower eyelid retraction, eyelid lag, GO-QOL score, CAS, and OSDI), showed no statistically significant difference between the groups before and after treatment, and CAS was measured only at baseline. Our study does not present the results as primary and secondary outcomes and does not generalize clinical improvement or deterioration as a whole. Instead, we observe each parameter (ocular motility, diplopia, MRD1, MRD2, eyelid aperture, levator function, proptosis, modified GO-QOL, and CAS) individually. Therefore, we do not have a percentage of general improvement in or deterioration of TED as such. The results in our study show a statistically significant improvement in both groups after 12 weeks for the parameters CAS, ocular motility, diplopia, and MRD1. Additionally, in the group treated with doxycycline, there were improvements in the parameters GO-QOL, MRD2, and levator function. Among the prior results, CAS, MRD2, and GO-QOL have clinical significance. The aforementioned differences in results may be due to differences in the study settings. While Pan Y. et al. [44] treated one group of patients with doxycycline and the other with a placebo, in our study, the test group received doxycycline and the control group did not receive any therapy. Another difference is that their participants had to meet and maintain euthyroid status for at least one month before enrollment, and most patients had a relatively inactive stage of TED with a CAS of less than 3. On the other hand, our study included patients who had a TED of less than 18 months, regardless of their euthyroid status, and the majority (82.9%) in the treated group and 46.3% in the untreated group had an active stage of the disease, with a CAS greater than or equal to 3. It is also possible that the different results were influenced by racial differences. The study by Pan Y. et al. [44] was conducted on an Asian population, while our study included an exclusively white population.

To the best of our knowledge, this is the first study of its kind in Europe. According to this study, TED is four to five times more likely to improve with the doxycycline treatment than spontaneously during 12 weeks when concerning MRD 2, CAS, and GO-QOL parameters. This conclusion indicates the need for additional evaluations of new therapeutic options for TED. 

This study has several limitations. A small number of subjects were included in this randomized clinical trial. Another limitation of this research study is the absence of a control group receiving a placebo, coupled with their lower baseline CAS compared to the study group. The follow-up time was short, so a longer course of follow-up of a larger sample of the population is needed in order to draw more confident conclusions. Not only should a longer period of treatment be provided, but also a delayed follow-up for weeks or months after the end of treatment. Higher doses’ safety and efficacy should also be further evaluated, and placebo-controlled, double-masked trials should be conducted. Based on this study, it is believed that doxycycline could be an effective treatment option not only for TED, but also for other inflammatory diseases of the orbit. 

Some of the evaluated parameters can be improved, such as the subjective assessment of diplopia which can be evaluated not only as present or absent, but quantified as no diplopia, intermittent, non-constant, and constant diplopia. Ocular motility should be measured by a perimeter and recorded in degrees instead of defining it only as performed or not performed. Answering in the Graves’ orbitopathy-specific quality of life (GO-QOL) questionnaire is modified in terms of yes or no answers, while it could be more detailed as in the original version of the questionnaire where there are three different answer options: (1) severely limited, (2) slightly limited, and (3) unlimited; therefore, interpretation and scoring are different.

As stated several times throughout this paper, a sub-antimicrobial dose of doxycycline works in patients with hyperthyroidism. We did not divide our patients into groups depending on the level of thyroid hormone disorders, which would make it possible to compare the activity between the groups and would also significantly contribute to a more detailed analysis and new knowledge in the future. Doxycycline is sometimes used to treat TED by reducing inflammation and improving symptoms [45]. It is believed to inhibit certain enzymes involved in inflammation. 

All of this would significantly contribute to the sensitivity of this study.

## Figures and Tables

**Table 1:** Patient characteristics at baseline.

		Total (*n* = 82) N (%)	Treated (*n* = 41) N (%)	Untreated (*n* = 41) N (%)	*p* Value *
Gender	Female	72 (87.8)	35 (85.4)	37 (90.2)	0.5023
	Male	10 (12.2)	6 (14.6)	4 (9.8)	
Age	Median (IQR)	50 (44–60)	50 (44–59)	52 (45–65)	0.272
	Range	20–77	20–77	26–77	
Smoking status	Positive	28 (34.1)	14 (34.1)	14 (34.1)	1
	Negative	54 (65.9)	27 (65.9)	27 (65.9)	

* Chi square test or Mann–Whitey test.

**Table 2:** Changes in measures over 12 weeks of study in treated and untreated patients.

Parameter		Timepoint	Treated (*n* = 41) N (%)	Untreated (*n* = 41) N (%)	*p* Value	
					Treated	Untreated
CAS	>=3	Baseline	34 (82.9)	19 (46.3)	<0.001 ^1^	0.003 ^1^
		Week 4	19 (46.3)	15 (36.6)		
		Week 12	6 (14.6)	9 (22)		
GO-QOL	QOL < 14	Baseline	20 (48.8)	14 (34.1)	0.0078 ^2^	0.125 ^2^
		Week 12	12 (29.3)	18 (43.9)		
Impaired ocular motility	Upgaze	Baseline	9 (22)	5 (12.2)	0.0151	0.039 ^1^
		Week 4	5 (12.2)	2 (4.9)		
		Week 12	4 (9.8)	1 (2.4)		
	Downgaze	Baseline	3 (7.3)	1 (2.4)	0.717 ^1^	1 ^1^
		Week 4	4 (9.8)	1 (2.4)		
		Week 12	4 (9.8)	1 (2.4)		
	Adduction	Baseline	10 (24.4)	4 (9.8)	0.018 ^1^	0.039 ^1^
		Week 4	10 (24.4)	8 (19.5)		
		Week 12	4 (9.8)	5 (12.2)		
	Abduction	Baseline	11 (26.8)	4 (9.8)	0.002 ^1^	0.368 ^1^
		Week 4	5 (12.2)	5 (12.2)		
		Week 12	3 (7.3)	3 (7.3)		
Diplopia	Present	Baseline	24 (58.5)	8 (19.5)	<0.001 ^1^	0.017 ^1^
		Week 4	15 (36.6)	8 (19.5)		
		Week 12	5 (12.2)	1 (2.4)		
MRD1 (mm)	Median (IQR)	Baseline	4 (3–5)	5 (4–5)	<0.0001 ^3^	0.0002 ^3^
		Week 4	5 (4–6)	5 (4–5)		
		Week 12	5 (4–5)	5 (4–5)		
MRD2 (mm)	Median (IQR)	Baseline	7 (6.8–8)	7 (6–9)	<0.0001 ^3^	0.1939 ^3^
		Week 4	6 (5.8–7.3)	7 (5–9)		
		Week 12	6 (5–7)	7 (5–9)		
Eyelid aperture (mm)	Median (IQR)	Baseline	11 (10–12.3)	12 (10.8–13)	0.0879 ^3^	0.8118 ^3^
		Week 4	11 (10.8–12)	11 (11–13)		
		Week 12	11 (10–12)	11 (10.8–13)		
Levator (mm)	Median (IQR)	Baseline	16 (13–18)	17 (14.8–19)	<0.0001 ^3^	0.1171 ^3^
		Week 4	17 (16–19)	18 (15.8–19.3)		
		Week 12	18 (16.8–19.3)	18 (16–18)		

^1^ Cochran’s Q test; ^2^ McNemar test; ^3^ Friedman test.

**Figure A1:**
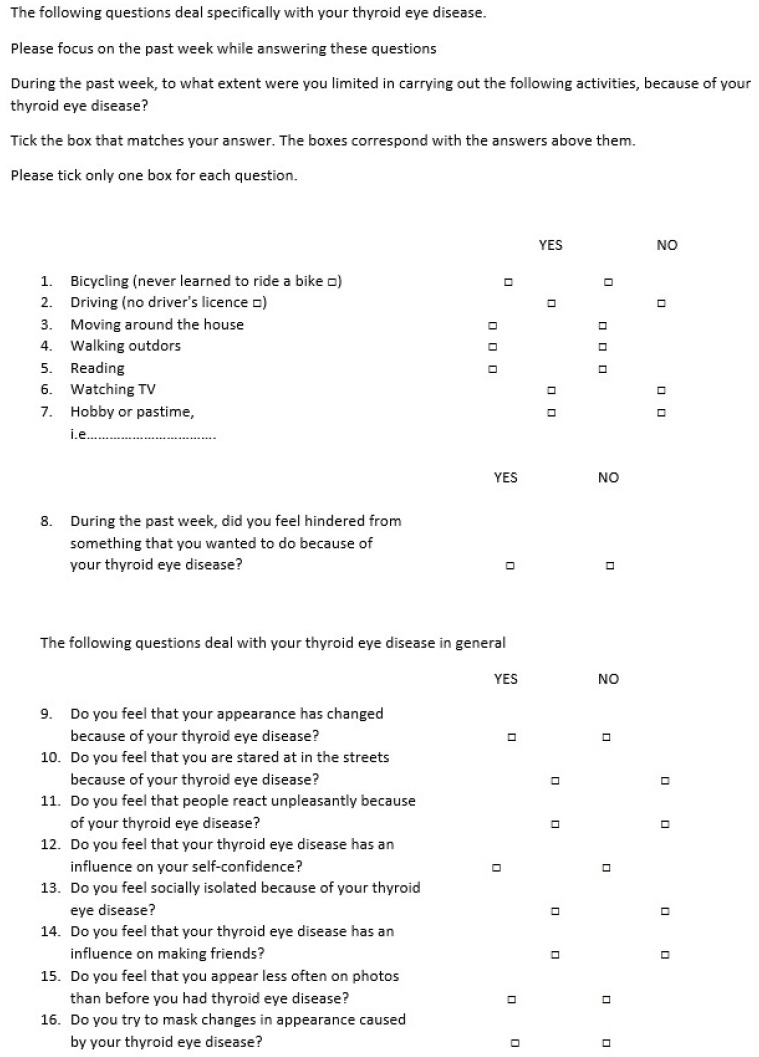
The modified GO-QOL questionnaire with yes and no answer options. The participant receives 1 point for the answer “YES” and 0 points for the answer “NO”. This questionnaire was downloaded from [46].

## Data Availability

The raw data are available upon reasonable request from the corresponding author.
